# Translational MicroRNA research in COVID-19: bridging in Silico prediction with clinical biomarker potential

**DOI:** 10.1007/s10238-025-02028-9

**Published:** 2026-01-21

**Authors:** Fathia Z. El Sharkawi, Marwa A. Mohamed, Karim Montasser, Mamdouh M. Mahdi, Hanaa B. Atya

**Affiliations:** 1 Biochemistry and Molecular Biology Department, Faculty of Pharmacy, Helwan University, P.O. Box 11795, Cairo, Egypt; 2 Department of Clinical Pathology, Faculty of Medicine, Helwan University, Cairo, Egypt; 3 Department of Internal Medicine and Nephrology, Faculty of Medicine, Helwan University, Cairo, Egypt

**Keywords:** MiRNAs, IL-6, HIF-1α, COVID-19

## Abstract

COVID-19 continues to evolve. Thus, new therapeutic alternatives are needed. Some in silico analyses, including the role of microRNAs, have shown promising results, but they are based on computational simulations and predictions, so their consistency with real-world results may vary. This study investigated the potential of three miRNAs (miR-196a-5p, miR-98-5p, and miR-27a-3p) obtained from published bioinformatics results as promising biomarkers for different aspects of COVID-19 (susceptibility, severity, and therapeutic possibilities). The current study found that, serum levels of these miRNAs were significantly elevated in COVID-19 patients compared to healthy controls, particularly during the third wave patients which is contrast to that reported in In silico study regarding miR-98-5p. Additionally, serum levels of inflammatory markers such as interleukin − 6 (IL-6) and hypoxia inducible factor- 1 α (HIF-1α) were higher in patients compared to healthy control. miR-196a-5p exhibited high sensitivity and specificity for COVID-19 diagnosis. Finally, we highlight the necessity of validating microRNAs in patient serum, as our findings show that their differential expression is robust and independent of the initial predictive model. This study effectively translates a bioinformatics hypothesis into a verifiable biomarker through experimental validation regardless of the contradictory findings between hypothesis and the actual results.

## Introduction

Severe acute respiratory syndrome coronavirus 2 (SARS-CoV-2) is the causative agent of the COVID-19 pandemic. The viral genome consists of a single positive-strand genomic RNA approximately 30 kb in length that codes all viral proteins [1, 2]. Infection is mediated by the spike protein, which facilitates viral entry into host cells by binding to human angiotensin-converting enzyme (ACE2), where transmembrane protease serine 2 (TMPRSS2) activates fusion of the virus and cell membranes [3, 4].

Infection is characterized by a wide range of symptoms, which can cause serious health consequences, such pneumonia and acute respiratory failure [5, 6]. Despite being largely a respiratory illness, COVID-19 can also cause non-pulmonary symptoms, such as neurological, gastrointestinal, renal, and other cardiovascular issues [7].

During the early stage of SARS-CoV-2 infection, lymphocytes are attacked, and their apoptosis is encouraged by the inflammatory response. Some symptoms subsequently appear when the quantity of virus particles increases. For example, the loss of oxygen diffusion ability can destroy the endothelium barrier. Interleukin-6 (IL-6), IL-2, and IL-7 are among the cytokines that increase systemic inflammation in patients with severe COVID-19. This inflammation affects distant organs and can lead to multiorgan failure and even death [8].

New variants of SARS-CoV-2 have emerged since its genome is prone to mutations, indicating that the virus is continuously evolving and challenging our therapeutic strategies. Different countries face three patterns of reported cases: the first wave occurs in spring (2020), the second wave occurs at the end of summer and autumn, and the third wave occurs at the beginning of 2021 [9]. In addition to the new variants that appeared in South Africa, Omicron (B.1.1.529) and Delta in India (lineage B.1.1617) [10] were identified. Delta was detected in October 2020 in India, and Omicron was detected in November 2021 [11].

MicroRNAs (miRNAs) are considered crucial players in host‒virus interactions [12]. They alter the host immune system against the virus, either by promoting viral replication or altering miRNA-mediated host gene regulation [13]. Host miRNAs interact with viral RNA by connecting to the 5′ UTR, 3′ UTR, and coding regions of the viral genome [14–16]. The effects of binding resulted in either inhibition or improvement in RNA viral translation [17]. Disruptions in miRNA regulation are usually accompanied by abnormal synthesis or secretion of miRNAs in cells or blood and have significant implications for various diseases, including cancers [18, 19], diabetes [20], cardiovascular diseases [21]. and virus-infected diseases [22].

SARS-CoV-2, like other coronaviruses, has been shown to regulate host miRNAs that can control viral proliferation and their spread [23]. There are possibilities that SARS-CoV-2 could also utilize virus-encoded miRNAs to infect the host for its own interest [24, 25]. COVID-19 infection is also characterized by an excessive and uncontrolled inflammatory response, which leads to the release of inflammatory cytokines. This process is termed the cytokine storm and leads to lung failure and death [26]. Several works have demonstrated that miRNAs are important regulators of the gene expression of these inflammatory genes [27–29] and could also be used against the COVID-19 cytokine storm.

miRNAs interact with SARS-CoV-2 via different mechanisms and therefore could be important therapeutic targets against COVID-19. On the other hand, there is already evidence that miRNAs could be used as effective antiviral agents. One example is the use of a miR-122 antagonist (Miravirsen) in the treatment of hepatitis C virus (HCV) infection [30, 31]. Many more examples of the use of miRNAs as antiviral agents are available [32, 33].

Fujii YR used the microRNA (miRNA) encoding sorting (METS) algorithm with artificial intelligence (AI) machine learning (MIRAI) analysis to reveal host and viral miRNAs along with their implications in associated functional pathways [34]. He predicted SARS-CoV-2 miRNA candidates from two stem loops in the 3’ terminus and from the 3’UTR because functional RNA viral miRNAs are encoded in the noncoding region of the 3’ end. To cohere SARS-CoV-2 miRNA candidates in the METS analysis, the seed paralog of viral miRNA candidates was searched in that of human host miRNAs. Cov-miR-1, Cov-miR-2, Cov-miR-4, Cov-miR-5 and Cov-miR-6 are the seed paralogs of miR-4310 (75% homologous), miR-4291 and miR-3611 (75%), miR-576-3p (88%), miR-27a/b-3p (75%), and miR-216a-3p and miR-29c-5p (75%), respectively. He reported that inflammatory IL-6 expression was directly upregulated by downregulation of the miR-98-5p hub. In contrast, a decrease in HIF-1α was observed. This is the first report showing that the etiology of COVID-19 is independent of host miRNAs and viral miRNAs [34].

On the basis of the data from Fujii YR’s study, the objective of the current study was to select specific miRNAs (miR-98-5p, miR-196a-3p, and miR-27a-3p) and measure their levels in the serum of COVID-19 patients to correlate these miRNAs with the inflammatory markers IL-6 and HIF-1α and predict that these miRNAs could serve as therapeutic targets or noninvasive biomarkers during COVID-19 infection.

## Patients and methods

### Study design

The present study included one hundred and fifty (150) subjects, who were classified into 100 clinically diagnosed COVID-19 patients and 50 healthy volunteers, who were matched for age, sex, and ethnicity, as controls. Patients were recruited from the inpatient department of the Quarantine Unit, Badr Hospital, Faculty of Medicine, Helwan University, Cairo, Egypt, from January 2021–January 2022.

Healthy controls (50 subjects) were recruited in early 2021, including 28 males and 22 females. The average age was 58 years (range 28–64). Controls were screened to exclude any history or clinical symptoms, any vaccination of COVID-19, other acute infections, or chronic inflammatory conditions (such as severe cardiovascular disease or active cancer) that could affect circulating microRNA levels. Additionally, they were not receiving treatment for any medical conditions.

Samples were collected with the written consent of the participants. The study was approved by the scientific research ethics committee of the faculty of pharmacy at Helwan University according to the regulations and recommendations of the Declaration of Helsinki (number 01H2021). The patient cohort was subclassified into three subgroups according to the disease period. The first group (from January to April 2021) included 35 patients, almost corresponding to the third wave (3rd wave) of the COVID-19 pandemic. The second group (from July to September 2021) included 40 patients, almost corresponding to the fourth wave (4th wave) of the COVID-19 pandemic. The third group (from December 2021 to January 2022) included 25 patients (the Omicron variant) whose Omicron variant was first reported to the WHO on 24 November 2021.

The inclusion criteria for this study were the use of hospitalized patients infected with SARS-CoV-2, which was confirmed by one of the following tests: a positive reverse transcriptase polymerase chain reaction (RT‒PCR); an antigen-based test from any respiratory, nasopharyngeal, saliva, or blood sample; or a radiological assessment via initial chest X-ray or CT scan.

The exclusion criteria were as follows: (i) suspected active uncontrolled bacterial, fungal, viral, or other infections (in addition to COVID-19) and (ii) active lung cancer or a history of lung cancer or any other lung disease. The control group must have had neither chronic respiratory disorders nor COVID-19 infection at the time of sample collection.

The medical data of all patients were recorded from patients’ files, including demographics and routine laboratory investigations, such as complete blood count (CBC), coagulation factor, and serum biochemical tests (including renal and liver function tests). Additionally, laboratory data, such as serum ferritin, D-dimer, and C-reactive protein (CRP) levels, were recorded to assess the severity of COVID-19, and the final outcomes, whether the patients survived or died, were recorded.

## Sample collection and preparation

Venous blood samples were collected from both control subjects and patients into serum-separating tubes, then centrifuged at 3000 rpm for 10 min. The sera were separated and aliquoted for the analysis of interleukin 6 (IL-6), hypoxia-inducible factor-1 (HIF), and selected miRNA extraction. Samples from COVID-19 patients were taken upon hospital admission, prior to starting any specific therapies (like corticosteroids, remdesivir, or high-flow oxygen). This was typically on the first day of ICU admission. All samples were stored at −80 °C for later analysis.

## Determination of serum IL-6 and HIF-1α levels

Serum levels of interleukin 6 (IL-6) (ELISA INNOVA, Biotech Co. LTD, Cat. no. In. Hu2192) and hypoxia-inducible factor-1α (HIF-1α) (ELISA INNOVA, Biotech Co. LTD, Cat. no. In. Hu2392) were measured in all subjects using ELISA, following the manufacturer’s instructions, with a microplate reader (INFINITE 50 PLUS).

### Total miRNA extraction

Serum total miRNAs were extracted with TRIzol^®^ reagent via the miRNeasy Mini Kit (QIAGEN, ID. 217184). The RNA purity and concentration were checked via a Nanodrop spectrophotometer.

### MicroRNA quantification via qRT‒PCR

The isolated miRNAs were reverse transcribed using the TaqMan™ MicroRNA Reverse Transcription Kit (Applied Biosystems™, Cat. No. 4366596). The cDNA used for qRT‒PCRs comprised 18.67 µl of PCR mix (1 µl of TaqMan™ Small RNA Assay (20x) for mir98-5p, mir196a-5p, and mir27a-3p, all from Applied Biosystems™, Cat. No. 4427975), along with 10 µl of TaqMan™ Universal Master Mix II (Cat. No. 440043) and 7.67 µl of nuclease-free water, totaling 20 µl. Serum levels of the selected miRNAs were measured using specific primers listed in Table 1, employing a Bio-Rad Thermal Cycler (MJ MINI™ Gradient Thermal Cycler, Singapore). MiRNA levels were normalized to mir16-5p (Cat. No. 4427975) under the following conditions: initial incubation at 95 °C for 10 min, followed by 40 cycles of denaturation at 95 °C for 15 s, annealing, and extension at 60 °C for 60 s. An automatic threshold option was used to acquire Ct values for each miRNA primer set.

## MiRNA expression calculations

The relative quantification of the assessed miRNAs was calculated as the expression of a housekeeping miRNA (miR-16-5p) in the same sample via the ΔΔCT equation.

ΔCT = CT assessed gene - CT reference gene (miR-16-5p).

ΔΔCT = ΔCT (patient sample) - ΔCT (healthy sample).

RQ (Relative Quantification) = 2^−(ΔΔCT)^.

**Table 1:** Primers used for the studied MiRNAs

Gene	Species	Sequence (Forward 5 to 3)	(Reverse 5 to 3)
Has-miR-16-5p	Human	CTTAAGAACCCTCCTTACTC	AAGCTACCCTAGGGGAAGGA
hsa-miR-27a-3p	Human	UUCACAGUGGCUAAGUUCCGC	GGAACUUAGCCACUGUGAAUU
hsa-miR-98-5P	Human	AACAATACAACTTACTACCTCA	GCGAGCACAGAATTAATACGAC
hsa-miR-196a-5P	Human	UAGGUAGUUUCUGUUGUUGGG	ATCTGGAGGAGAAGGGAAGG

### Statistical analysis

Statistical analysis was performed using GraphPad Prism 9.0.0 (GraphPad Software, San Diego, CA). Normality of the data was assessed with the Kolmogorov-Smirnov and Shapiro-Wilk tests. As most data was non-normally distributed, nonparametric tests were used for all analyses. Descriptive statistics are presented as means, medians, and standard errors of the mean (SEM). The nonparametric Mann-Whitney U test and Kruskal-Wallis H test were utilized to compare quantitative variables and fold changes in the expression of selected miRNAs. Spearman bivariate correlation analysis assessed the strength of linear relationships between serum miRNA expression levels and other clinical parameters. The ∆CT values were expressed as the median (ME) and plotted as box-whisker plots, which represent the median, minimum and maximum values of determined miRNA expression and were used for the receiver operating characteristic (ROC) curve to differentiate between the selected miRNAs and evaluate the predictive diagnostic potential of miRNAs. via Med Cala 9.3.9.0 (MedCala, Mariakerke, Belgium). All hypothesis tests were two-tailed, with p-values < 0.05 considered statistically significant.

## Results

### Demographic and clinical data

The demographic characteristics of all patients, including the number of patients in each wave, biochemical parameters, and clinical parameters, are documented in (Table 2**)**.

The patient group comprised 51% males and 49% females, whereas the control group consisted of 56% males and 44% females. There was no significant difference in the distribution of sexes between the patients and the healthy controls (*P* > 0.05). The average age of the patients was 61.9 ± 2.3 years, which was not significantly different from that of the controls (58.7 ± 2.5 years) (*P* > 0.05).

**Table 2:** Demographic and clinical data of COVID-19 patients

Parameters (Mean ± SEM)	3rd wave *N* = 35	4th wave *N* = 40	Omicron wave *N* = 25
**Gender**	Male = 22 Female = 13	Male = 16 Female = 24	Male = 13 Female = 12
**Total leukocyte count (TLC)**	12.18 ± 1.45	8.82 ± 1.3	7.98 ± 0.65
**Red Blood Cells (RBCs)**	4.586 ± 0.1671	4.355 ± 0.2058	3.99 ± 0.4801
**Lymphocytes**	8.864 ± 1.465	22.88 ± 4.468	18.68 ± 2.47
**Hemoglobin (HB)**	12.78 ± 0.53	12.02 ± 0.44	12.31 ± 0.44
**Hematocrit (HCT)**	38.5 ± 1.3	38.15 ± 1.4	37.85 ± 1.5
**Mean Corpuscular Volume (MCV)**	84.27 ± 1.4	85.27 ± 1.48	84.6 ± 1.36
**Mean Corpuscular Hemoglobin (MCH)**	27.89 ± 0.59	26.26 ± 0.43	27.36 ± 0.44
**Mean Corpuscular Hemoglobin Concentration (MCHC)**	33.04 ± 0.26	34.04 ± 0.46	32.04 ± 0.18
**Red Cell Distribution Width (RDW)**	15.08 ± 0.56	14.8 ± 0.96	15.28 ± 0.76
**Platelets (PLT)**	251.4 ± 27.18	246.8 ± 66.51	193.6 ± 23.31
**Aspartate Transferase (AST) (IU/L)**	45.18 ± 6.630	40.08 ± 8.455	52.75 ± 9.625
**Alanine Aminotransferase (ALT) (IU/L)**	34.32 ± 3.752	40.64 ± 4.585	30.58 ± 4.712
**Albumin(g/dl)**	2.6 ± 0.17	2.7 ± 0.25	3.2 ± 0.18
**Creatinine (mg/dl)**	1.358 ± 0.1893	1.184 ± 0.1352	1.286 ± 0.2740
**Urea (mg/dl)**	57.58 ± 9.293	54.06 ± 7.265	81.60 ± 14.43
**D. Dimer (ng/ml)**	33.5 ± 1.5	33.5 ± 1.8	23.5 ± 2.5
**C-Reactive Protein (CRP) (ng/ml)**	61.43 ± 13.19	122.4 ± 19.2	66.48 ± 11.2
**International Normalized Ratio (INR)**	1.190 ± 0.02	1.160 ± 0.012	1.130 ± 0.017
**Ferritin (ng/ml)**	1189 ± 222.4	1229 ± 202.4	889 ± 102.6
**Outcome**	12 Improved 23 Died	10 Improved 30 Died	24 Improved 1 Died

### Serum levels of interleukin-6 (IL-6)

**Fig. 1:**
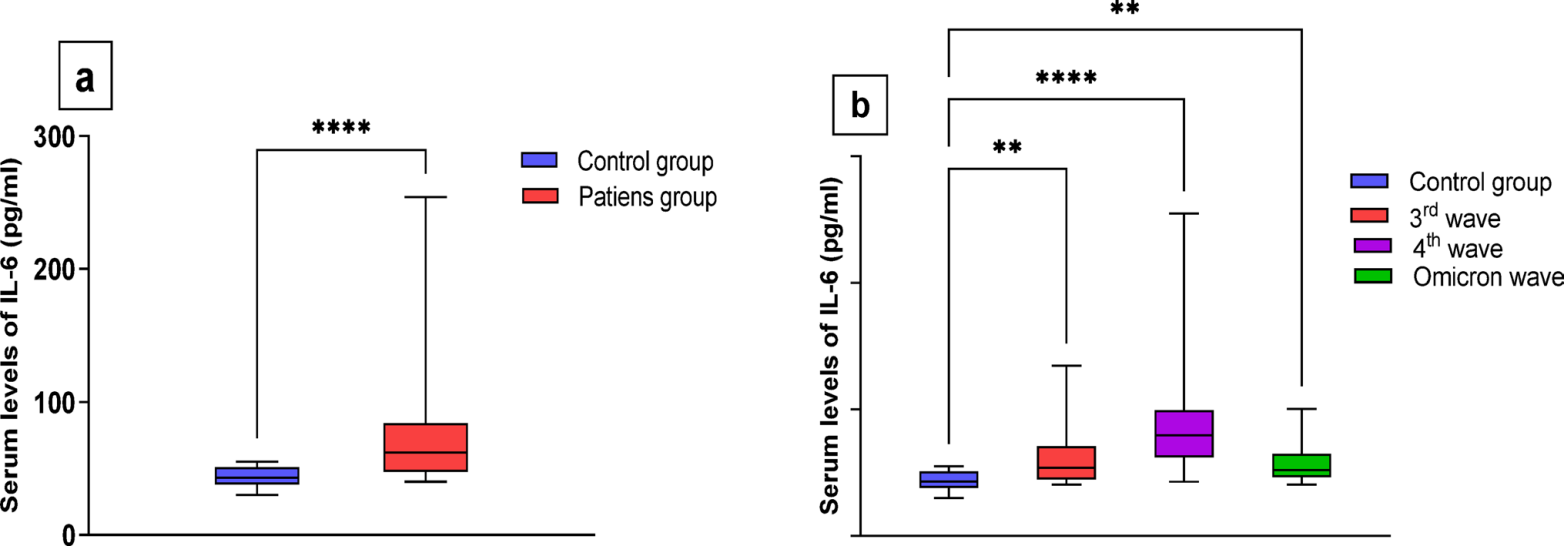
**a**- Serum levels of IL-6 in COVID-19 patients and control groups using Mann-Whitney U test. **b**- Serum levels of IL-6 in patients’ subgroups and control groups using and Kruskal-Wallis H test followed by Multiple comparison test. ** *P* ≤ 0.01, **** *P* ≤ 0.0001

The mean serum level of IL-6 was 68.8 ± 3.2 pg/ml in all COVID-19 patients, while it was 44.1 ± 1.1 pg/ml in the control group. IL-6 levels were significantly higher in patients group, with a median of 68.00 compared to a median of 43.00 in controls (*p* ≤ 0.0001), as illustrated in Fig. 1a. Additionally, serum IL-6 levels were evaluated in patient subgroups during the 3rd wave, 4th wave, and Omicron wave, showing significant differences (61.4 ± 3.9 pg/ml for the 3rd wave, 84.1 ± 6.4 pg/ml for the 4th wave, and 58.8 ± 3.4 pg/ml for the Omicron wave), with corresponding medians of 53.83, 79.73, and 52.05, respectively **(**Fig. 1b).

### Hypoxia-inducible factor-1α (HIF-1α)

**Fig. 2:**
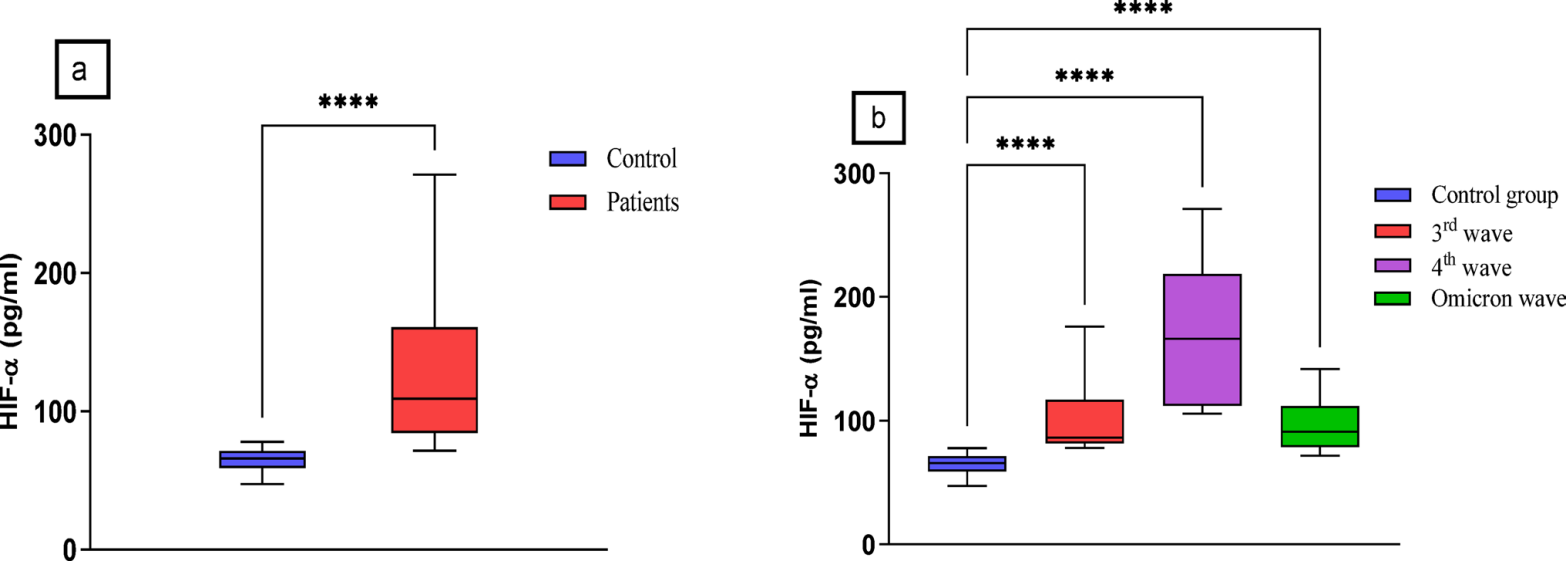
**a**- Serum levels of HIF-1α in COVID patients and control groups using Mann-Whitney U test. **b**- Serum levels of HIF-1α in patients’ subgroups and control groups using and Kruskal-Wallis H test followed by Multiple comparison test. **** *P* ≤ 0.0001

Serum levels of HIF-1α were significantly higher in all patients compared to controls, with values of 127.2 ± 5.6 pg/ml and 64.9 ± 1.3 pg/ml, respectively (*p* < 0.0001). The median for patients was 109.2, while controls had a median of 65.75 (Fig. 2a). Additionally, there were statistically significant differences in HIF-1α levels among the three patient groups compared to the control group. The 4th wave group showed the highest levels of HIF-1α (170.2 ± 9.1 pg/ml, median = 166.3), followed by the 3rd wave (101.5 ± 5.1 pg/ml, median = 86.43) and the Omicron wave (94.8 ± 3.9 pg/ml, median = 91.15), as described in Fig. 2b.

### Serum levels of the selected miRNAs

**Fig. 3:**
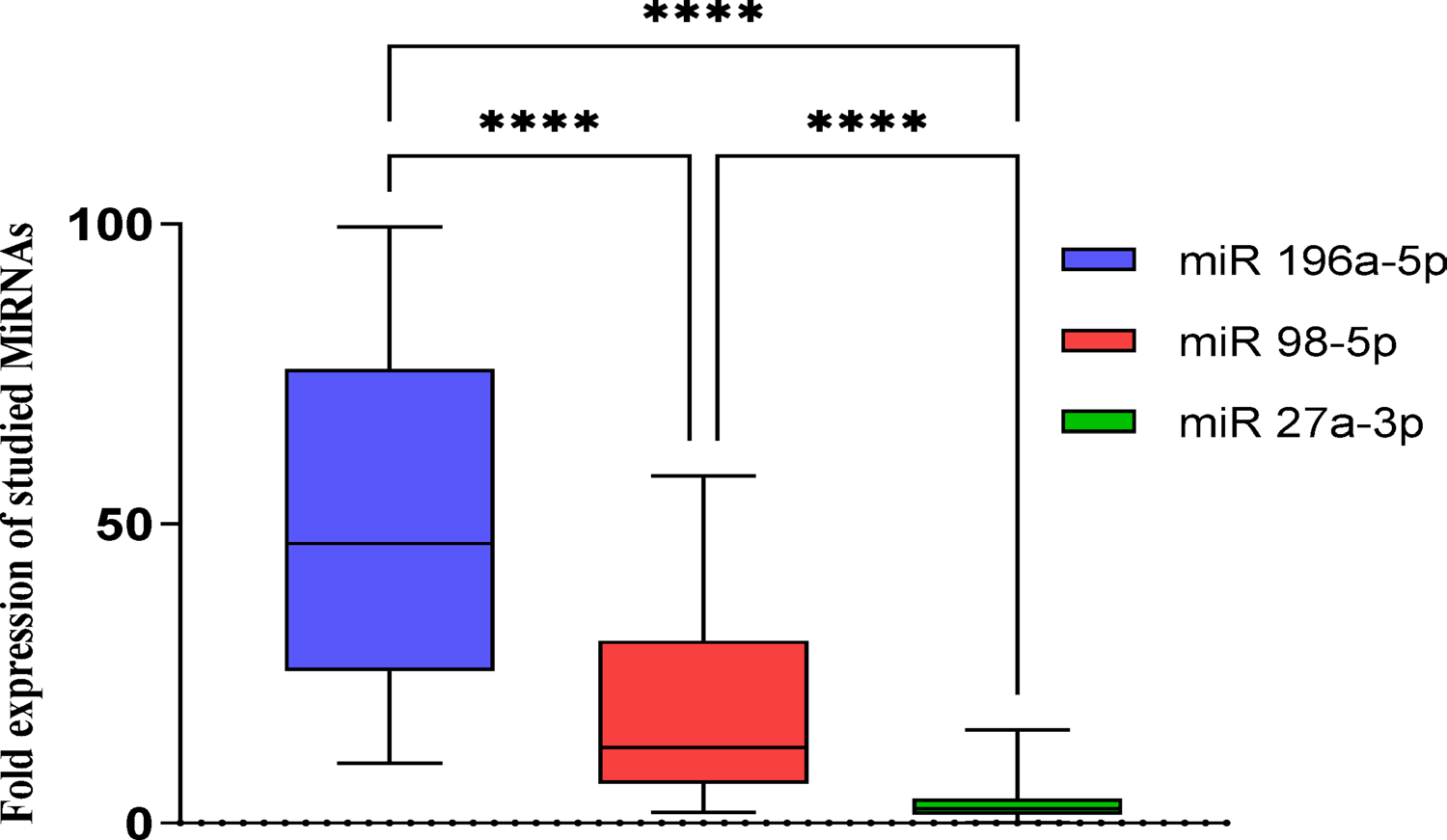
Box-Whisker plots of the median miRNA’s expressions, expressed as fold change from the control group, comparison between miRNAs was made using Kruskal-Wallis H test followed by Multiple comparison test. **** *P* ≤ 0.0001

All examined miRNAs were significantly expressed in patients across different COVID-19 waves compared to the control group. The expression level of miR-196a-5p was particularly high at 51.6 ± 2.9-fold, in contrast to miR-98-5p (18.6 ± 1.7-fold) and miR-27a-3p (3.1 ± 0.3-fold). The descending order of miRNA expression levels was 3rd wave > 4th wave > Omicron wave, with medians of 46.73, 12.61, and 2.43, respectively (*p* ≤ 0.0001) as shown in Fig. 3.

**Fig. 4:**
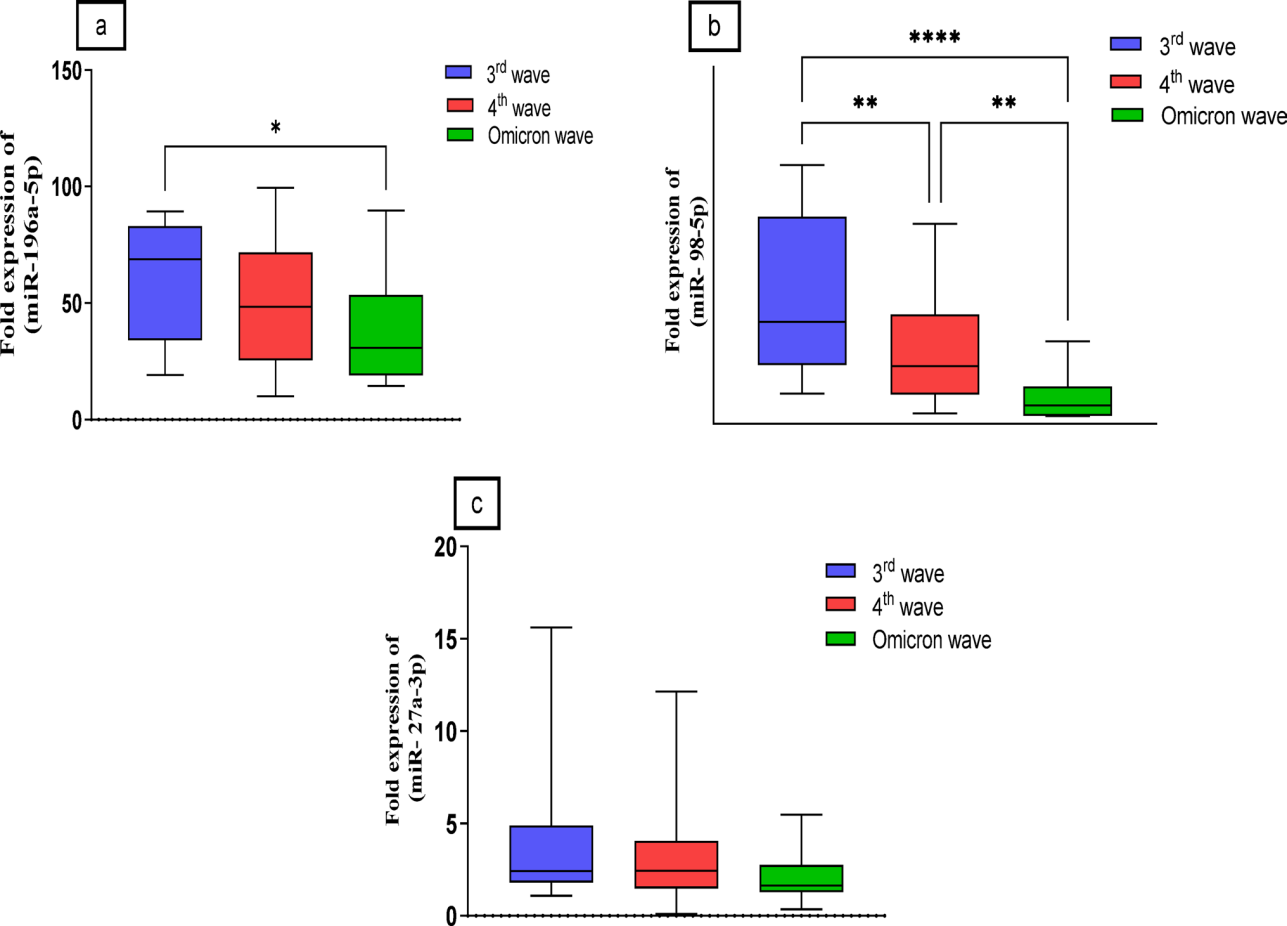
Box-Whisker plots of the median miRNA’s expressions in different subgroups, **a**: Expression levels of miR-196a-5p, **b**: Expression levels of miR-98-5p, **c**: Expression levels of miR-27a-3p, expressed as fold change from control group. comparison between miRNAs was made using Kruskal-Wallis H test followed by Multiple comparison test. *P ≤ 0.05, ** *P* ≤ 0.01, **** *P* ≤ 0.0001

For miR-196a-5p, the 3rd wave exhibited the highest expression level (60.4 ± 4.4-fold, median = 68.85), followed by the 4th wave (50.5 ± 4.9-fold, median = 48.8) and the Omicron wave (39.28 ± 5.6-fold, median = 30.75) (*p* ≤ 0.05). While miR-98-5p demonstrated an expression of 29.52 ± 3.2-fold with a median of 22.9 in the 3rd wave, significantly higher than the 4th wave (16.46 ± 2.2-fold, median = 13.0, *p* ≤ 0.01) and the Omicron wave (5.6 ± 0.9-fold, median = 4.13, *p* ≤ 0.0001). Lastly, miR-27a-3p showed no significant differences across the three waves, with expressions of 3.7 ± 0.5-fold (median = 2.42) in the 3rd wave, 3.2 ± 0.4-fold (median = 2.44) in the 4th wave, and 2.02 ± 0.3-fold (median = 1.6) in the Omicron wave (Fig. 4a, b, c).

**Fig. 5:**
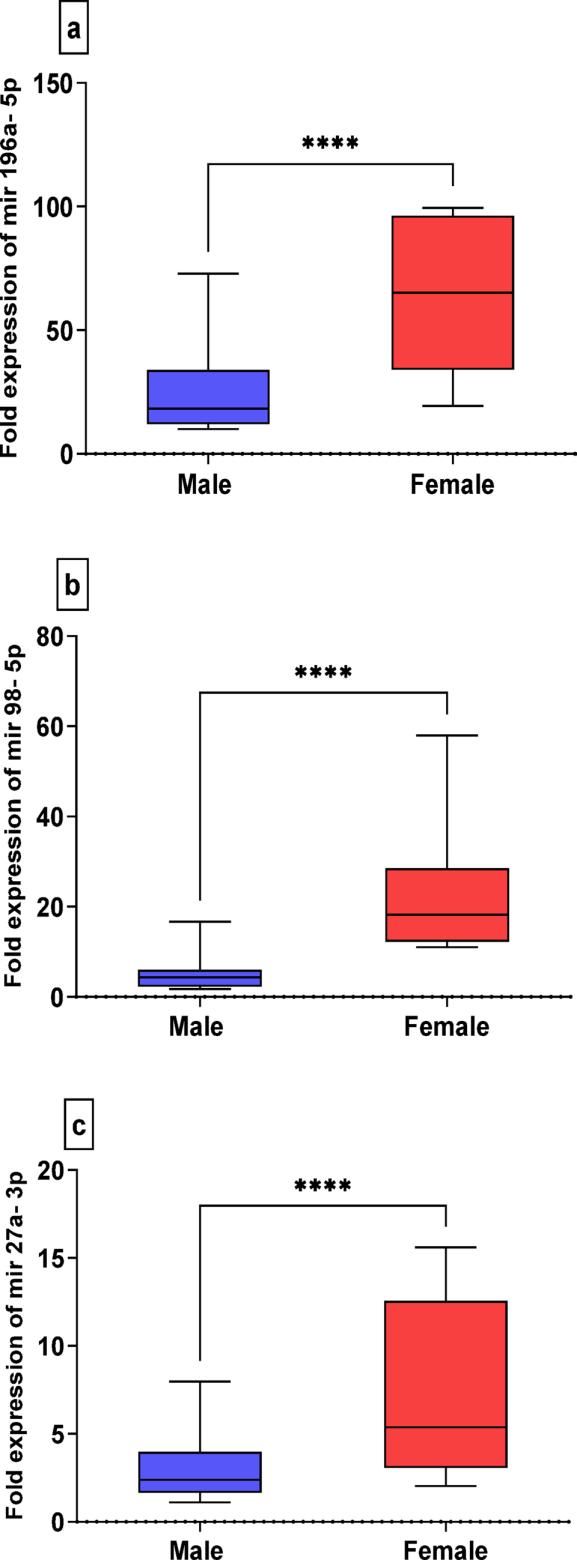
Box-Whisker plots of the median miRNA’s expressions in male and female COVID patients, **a**: Expression levels of miR-196a-5p, **b**: Expression levels of miR-98-5p, **c**: Expression levels of miR-27a-3p, expressed as fold change from control group, using Mann-Whitney U test, **** *P* ≤ 0.0001

The analysis of sex differences in the expression levels of the selected miRNAs revealed significant female-biased expression in COVID-19 patients, as shown in Fig. 5a, b, c. MiR-196a-5p was upregulated in female patients (62.9 ± 4.8, median = 65.25) compared to male patients (25.4 ± 2.6, median = 18.27) (Fig. 5a). Similarly, both miR-98-5p and miR-27a-3p were expressed at higher levels in females (23.1 ± 2.2, median = 18.2 and 7.1 ± 0.6, median = 5.4, respectively) than in males (5.7 ± 0.6, median = 4.3 and 3.12 ± 0.3, median = 2.4, respectively) (Fig. 5b, c).

**Fig. 6:**
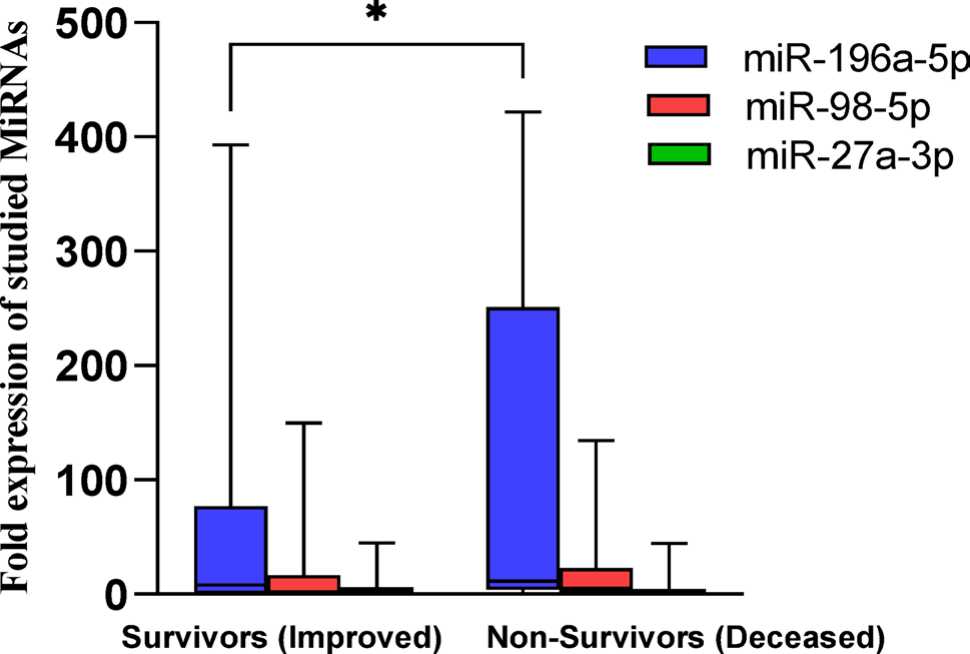
Box-Whisker plots of the median expression rates of the miRNAs in serum of all COVID-19 patients according to their outcome (Improved, Deceased) using Kruskal-Wallis H test followed by Multiple comparison test, * *P* ≤ 0.05

After stratifying COVID-19 patients by outcomes into survivors (improved, *n* = 46) and non-survivors (deceased, *n* = 54), our study found a significant increase in miR-196a-5p levels in deceased patients compared to improved ones (109.2 ± 21.4-fold, median 11.5 vs. 74.4 ± 20.8-fold, median 7.9, *P* < 0.05). In contrast, the other miRNAs did not show significant differences: miR-98-5p levels were 18.5 ± 4.9-fold (median 5.1) in deceased patients versus 17.2 ± 5.5-fold (median 1.9) in improved patients, and miR-27a-3p levels were 7.6 ± 2.1- fold (median 1.8) in deceased patients compared to 6.9 ± 1.7- fold (median 1.5) in improved patients as illustrated in (Fig. 6**)**.

### Receiver operating characteristic (ROC) curve for estimation of diagnostic efficiency

**Fig. 7:**
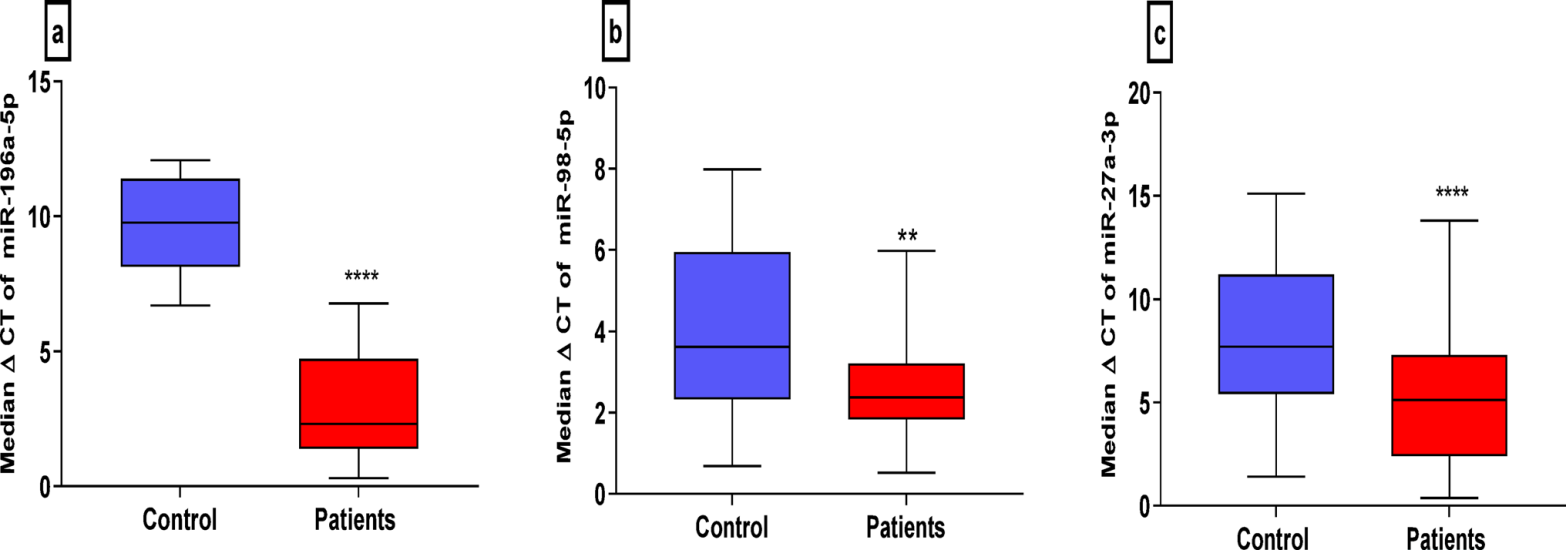
Box-Whisker plots of the median expression rates of the miRNAs in serum of all patients and control expressed as median ΔCT values. Higher ΔCT values stand for lower miRNA expression. **a**: Expression levels of miR-196a-5p, **b**: Expression levels of miR-98-5p, **c**: Expression levels of miR-27a-3p using Mann-Whitney U test, ** *P* ≤ 0.01, **** *P* ≤ 0.0001

A receiver operating characteristic (ROC) curve was built from the ΔCT values of the selected miRNAs, which were plotted via box‒whisker plots to distinguish normal subjects from COVID-19 patients and are expressed as median ΔCT values. Higher ΔCT values indicate lower miRNA expression **(**Fig. 7a, b, c**)**.

**Fig. 8:**
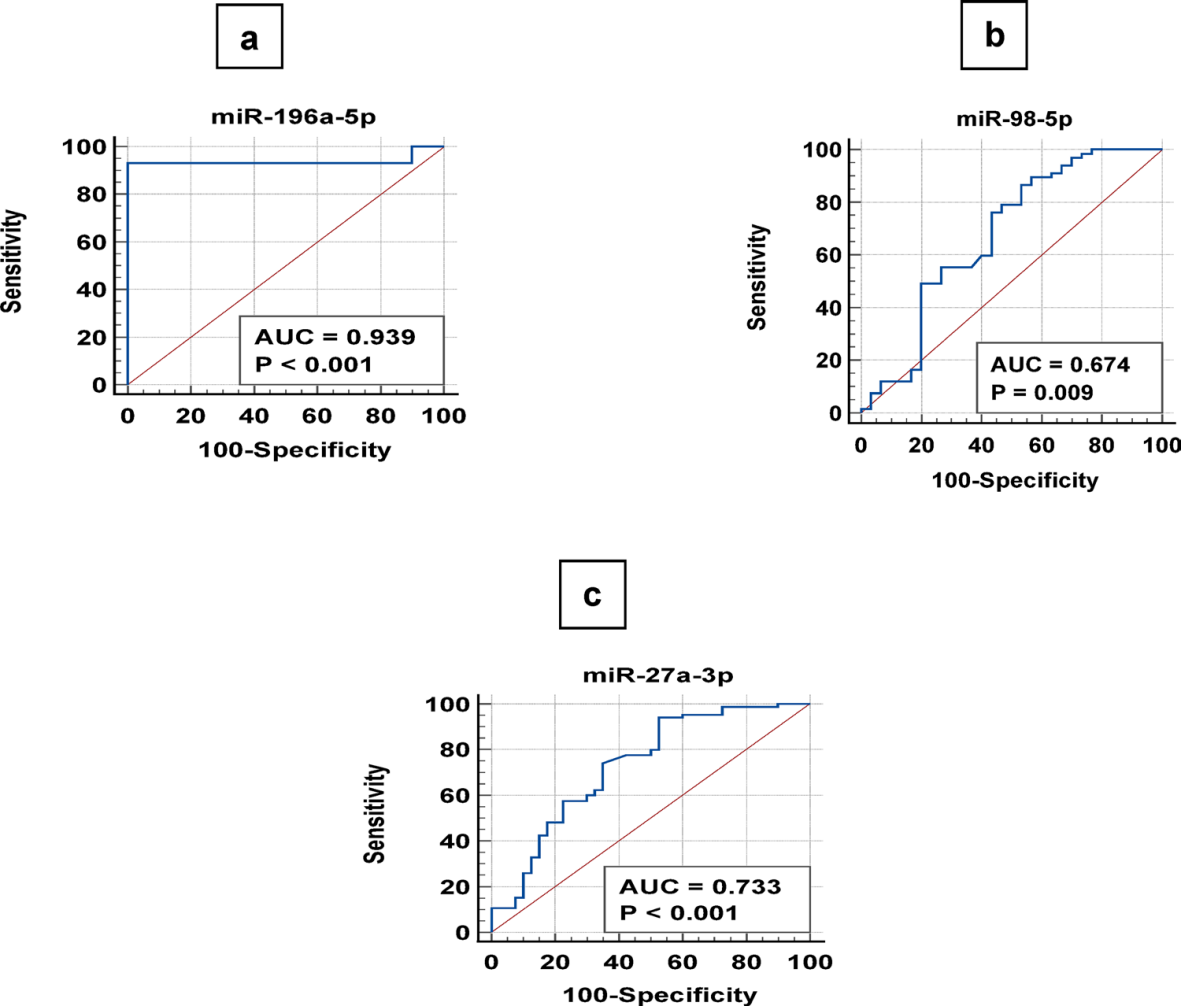
Receiver operating characteristic (ROC) curve for miRNAs based on the RT-qPCR data. The diagram is a plot of the sensitivity (true-positive rate) vs. 100-specificity (false-positive rate). AUC is the area under the curve. **a**: ROC curve for miR-196a-5p, **b**: ROC curve for miR-98-5p, **c**: ROC curve for miR-27a-3p

A ROC curve was generated, and the AUC was defined. All the Biomarkers yielded significant AUC values. The upregulated miRNAs yielded adequate performance and confirmed that all three miRNAs were of significant diagnostic value for COVID-19 infection. The overall sensitivity, specificity, and AUC values are listed in (Table 3**)** and shown in Fig. 8a, b, c.

**Table 3:** Statistical results of the comparison between the patient and control groups for the investigated MiRNAs based on their ∆CT values

miRNAs	Fold change	AUC	Sensitivity	Specificity	*P* value
miR-27a-3p	3.1	0.733	94.12	47.5	< 0.001
miR-98-5p	18.6	0.674	86.6	46.7	= 0.009
miR-196a-5p	51.6	0.939	93.2	100.0	< 0.001

AUC: Area under the curve

### Correlations between the selected miRNAs and several biochemical and clinical parameters

**Fig. 9:**
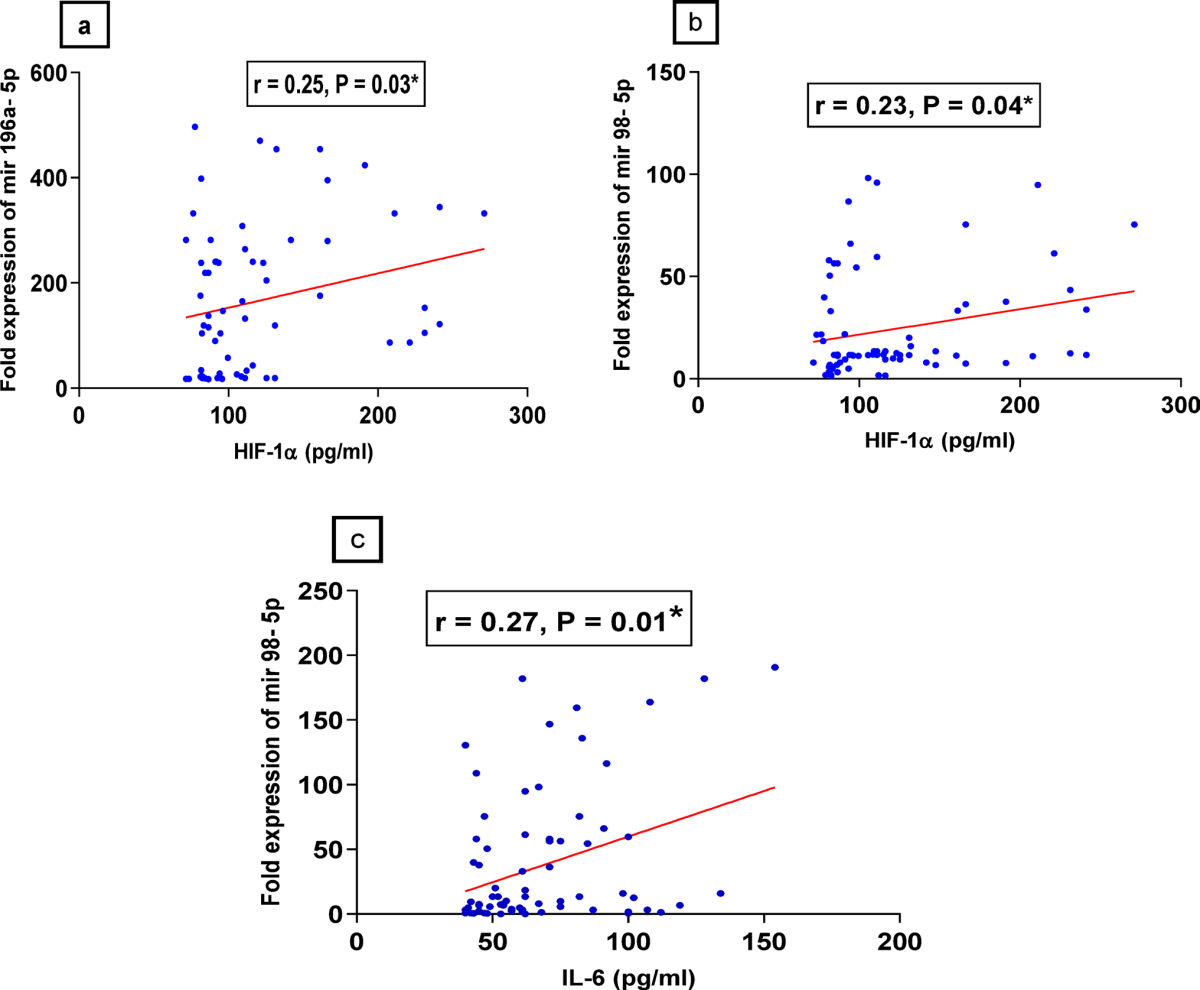
Correlation between serum levels of selected miRNAs and some biochemical parameters. **a**: correlation between the serum levels of miR-196a-5p and the levels of HIF-1α, **b**: correlation between the serum levels of miR-98-5p and the levels of HIF-1α, **c**: correlation between the serum levels of miR-98-5p and the levels of IL-6. r: Spearman coefficient, * *P* < 0.05, ** *P* < 0.01

There was a direct correlation between the serum levels of miR-98-5p and the levels of IL-6 and HIF-1α. However, miR-196a-5p was positively correlated with HIF-1α only. On the other hand, a significant association between D-dimer and miRNA-196a-5p was found **(**Table 4**and** Fig. 9a, b, c**)**.

**Table 4:** Correlations between the serum levels of the studied MiRNAs and several biochemical and clinical parameters

Parameters	miR-27a-3p	miR-98-5p	miR-196a-5p
Age ≤ 60 ˃ 60 P value	15.22 ± 2.215, *n* = 40 5.038 ± 1.473, *n* = 46 0.1181	10.48 ± 1.845, *n* = 45 7.603 ± 1.803, *n* = 46 0.2683	109.6 ± 10.82, *n* = 48 17.12 ± 2.308, *n* = 42 < 0.0001***
Gender Male Female P value	3.12 ± 0.3 7.1 ± 0.6 0.0001***	5.7 ± 0.64 23.05 ± 2.2 0.0001***	25.4 ± 2.6 62.9 ± 4.8 0.0001***
COVID-19 waves 3rd wave group 4th wave group Omicron wave group	3.7 – fold higher 3.2 – fold higher 2.02 – fold higher	29.5 – fold higher 16.5 – fold higher 5.6 – fold higher	60.4 – fold higher 50.5 – fold higher 39.3 – fold higher
D-dimer (ng/ml)	*r* = 0.18, *p* = 0.3	*r* = 0.22, *p* = 0.3	*r* = 0.5, *p* = 0.0001***
CRP (ng/ml)	*r* = 0.01, *p* = 0.9	*r* = − 0.17, *p* = 0.4	*r* = 0.09, *p* = 0.07
Ferritin (ng/ml)	*r* = 0.24, *p* = 0.26	*r* = − 0.06, *p* = 0.8	*r* = 0.28, *p* = 0.35
IL-6 (pg/ml)	*r* = − 0.02, *p* = 0.8	*r* = 0.27, *p* = 0.018*	*r* = 0.05, *p* = 0.69
HIF − 1α (pg/ml)	*r* = − 0.06, *p* = 0.6	*r* = 0.23, *p* = 0.04*	*r* = 0.25, *p* = 0.03*

CRP: C- Reactive protein, IL-6: Interleukin − 6, HIF − 1 α: Hypoxia inducible factor 1 alpha. r: Spearman coefficient

## Discussion

The specific treatments for COVID-19 are still being explored [35]. Some researchers are utilizing bioinformatics studies to analyze the characteristics and pathogenesis of COVID-19, which may help in identifying novel therapeutic targets for the development of effective drugs. However, further research and testing are necessary to translate these findings into practical solutions for managing and preventing COVID-19 [36]. Acute inflammation and viral infection mechanisms in COVID-19 have been shown in host and viral miRNA networks [34]. SARS-CoV-2 infection induced IL-6 upregulation via downregulation of the miR-98-5p hub, and hypoxia was also induced by protein HIF-1α suppression by viral miRNAs and miR-196a-5p [34].

In the present study, the authors selected several miRNAs from the Fujii YR study (miR-98-5p, miR-196a-5p, and miR27-3-p) to perform more detailed in vivo validations and investigate their biological significance in the pathogenesis of COVID-19.

The increased serum levels of IL-6 in the COVID-19 patients in this study, particularly in the 3rd and 4th waves, are due to cytokine-releasing syndrome (CRS), which occurs in patients with severe COVID-19 and results in high levels of IL-6, IL-1β, and monocyte chemoattractant protein-1 (MCP-1) [37, 38]. IL-6, which is a central mediator of fever and acute phase inflammation, is secreted from macrophages, including alveolar macrophages, T and B lymphocytes, and fibroblast endothelial cells [39]. It has been suggested that cytokine storms caused by IL-6 dysregulation may be the cause of lung injury in COVID-19 patients [38]. The IL-6-inflammatory response is closely associated with metabolic disorders in patients with viral infection. Additionally, high serum levels of IL-6 in patients with SARS-CoV-2 are released into the liver via the bloodstream and subsequently induce the expression of several acute phase proteins, such as C-reactive protein (CRP) [40]. Moreover, IL-6 is involved in the regulation of serum iron and zinc levels by regulating their transporters [40]. In addition, the level of IL-6 is elevated in critically ill COVID-19 patients (4th and 3rd waves in the present study); in this case, many pathogenic T cells and inflammatory monocytes that secrete high levels of IL-6 may enter pulmonary circulation and trigger an inflammatory storm, leading to immune dysfunction and extensive injury, resulting in multiple system organ failure and even high mortality [40].

The results of the present study demonstrated that the increased serum levels of HIF-1α in COVID-19 patients were opposite to those reported in the Fujii study [34] but matched those reported in a recent study carried out on human cell lines infected with SARS-CoV-2 [41]. It has been suggested that the ORF3a protein of SARS-CoV-2 can stimulate HIF-1α production by damaging mitochondria and increasing mitochondrial reactive oxygen species (Mito-ROS). Consequently, HIF-1α promotes viral infection/replication and exacerbates inflammatory responses [41, 42]. Infection with SARS-CoV-2 in pulmonary tissues impairs gas exchange, leading to systemic hypoxia and increased glycolysis in endothelial cells and immune cells by stabilizing hypoxia-inducible factor-1 (HIF-1) [35, 43]. Notably, lactate can induce the activation of hypoxia-inducible factor-1 (HIF-1), which further enhances aerobic glycolysis and promotes SARS-CoV-2 infection and replication [43].

As previously mentioned, SARS-CoV-2 pathogenesis starts with specific recognition of ACE2 on the surface of ACE2-positive cells, including lung cells and capillary endothelium. Therefore, these cells are infected by the virus, followed by inflammation and hypoxia, which induce HIF-1α transcriptional activity [44]. HIF-1α activation can contribute to the cytokine storm by promoting the activation and stabilization of immune cells such as macrophages and neutrophils, causing the production of high levels of inflammatory cytokines and leading to an overwhelming inflammatory response [44]. Hence, HIF-1α, a crucial factor in response to the hypoxic microenvironment at the site of inflammation, acts as a ‘master regulator’ in phagocytes. Therefore, it can improve inflammatory responses by increasing the survival of these immune cells and their recruitment to sites of inflammation. Therefore, HIF-1α inhibition via pharmacological strategies might provide a new approach to aid in the treatment of patients affected by COVID-19. Furthermore, in addition to the probable immune modulatory effect of HIF-1α, this transcription factor has a positive impact on the autophagy process. Therefore, because autophagy recruits SARS-CoV-2 to host cells to increase their proliferation and progression, HIF-1α inhibition is another way to prevent viral infection [45].

With respect to the serum levels of the selected miRNAs, our study revealed associations between higher serum levels of miR-196a-5p, miR-98-5p, and miR-27a-3p and female sex, especially in the 3rd wave of COVID-19 patients.

In addition to the global waves driven by alpha, beta, delta, Omicron, and other notable variants, the third COVID-19 wave in Egypt may be driven by the circulation and spread of the beta and delta variants. The beta variant is known for its increased transmissibility and partial immune evasion, which likely contributed to its spread and impact in some regions [46]. Similarly, the delta variant has become the dominant strain in many parts of the world due to its high transmissibility and potential for more severe illness than previous variants [46].

The 4th wave in Egypt may be driven by Delta Plus variants known as AY.1 (B.1.617.2.1) and AY.2 (B.1.617.2.2), as they became variants of concern (VOCs) in summer 2021 globally. The Delta Plus variant has increased transmissibility, causes stronger binding to receptors of lung cells, and has the potential to reduce the monoclonal antibody response. Although this variant has a greater affinity for the mucosal lining in the lungs, it may not lead to serious and fatal illness compared with previous variants [46].

In addition, our study revealed that the serum levels of the selected miRNAs were lower during Omicron waves than during other COVID-19 waves. This agrees with the study by Antonelli et al. in 2022, which demonstrated that the Omicron variant may induce a less severe acute illness profile and be closely monitored compared with previous variants [47].

The increased serum levels of miR-196a-5p in our study were directly correlated with the levels of HIF-1α and D-dimer. Some studies have reported that miR-196a-5p is involved in lung injury and the growth of cancer cells, such as lung cancer and squamous cell carcinoma cells, and that its serum level is increased in non-small cell lung cancer (NSCLC) patients and is positively correlated with advanced tumor stage and positive lymph node metastasis [48, 49]. In addition, the serum level of miR-196a-5p is elevated in autoimmune diseases associated with SARS-CoV-2 infection, such as inflammatory bowel disease (IBD) [49].

Furthermore, a study by Li et al. revealed that miR-196a-5p is highly expressed in tumor-associated macrophages (TAMs) and the exosomes released by TAMs. The deletion of miR-196a-5p in TAMs significantly prevents lung cancer metastasis both in vitro and in vivo [50].

Other studies, such as Diallo et al., reported that miR-196a-5p was downregulated during the early stage (24 h postinfection) and was capable of being related directly to the viral genome, specifically through ORF1(a, b) and ORF2 (corresponding to the spike protein), and the suppression of this miRNA might be part of the viral strategies for better replication and escape from the host’s defenses [51].

The significant direct correlation between miR-196a-5p and D-dimer levels in our study may be explained as, miR-196a-5p regulates inflammatory cytokines such as IL-6, which activate endothelial cells and increase oxidative stress through excessive mitochondrial reactive oxygen species (mtROS) production, exacerbating endothelial dysfunction [52, 53].Furthermore, D-dimer levels can rise due to inflammation, especially lung inflammation caused by microbes, which are relevant in COVID-19. In severe cases of COVID-19, various activation events—such as inflammation, hemostasis, and fibrinolysis—contribute to increased D-dimer levels. Thus, miR-196a-5p may indirectly influence D-dimer levels by promoting pro-inflammatory cytokine production, like IL-6, leading to heightened oxidative stress in endothelial cells and consequently elevated D-dimer levels during COVID-19 [54].

The most notable finding is the significantly higher relative expression of miR-195a-5p in the deceased patient compared to the improved ones (approximately 109-fold vs. 74-fold, respectively). This overexpression indicates a strong association with poor prognosis and disease severity. miR-195a-5p is involved in regulating pathways related to cell survival, apoptosis, and inflammation across various disease models. Its considerable upregulation in non-survivors may reflect a failure to resolve inflammation or an acceleration of tissue damage, highlighting its potential as a negative prognostic marker. This aligns with findings by Xin H et al., who noted that high miR-196a-5p expression correlates with poor prognosis and increased disease severity, particularly in various cancers, where it often functions as an oncogene [55].

The serum level of miR-98-5p in the present study was found to be increased in COVID-19 patients and was directly correlated with the serum levels of both IL-6 and HIF-1α which is converse the results obtained from in silico study. Several studies have reported that miR-98-5p can target STAT3 and inhibit the IL-6/STAT3 signaling pathway, controlling proinflammatory cytokines in many inflammatory and autoimmune diseases and other IL-6-mediated diseases [56, 57].

A computational study conducted by Hosseini Rad et al. revealed that miR-98, among other human miRNAs, has the potential to bind to SARS-CoV-2 [58]. Moreover, experimental research conducted by other researchers confirmed that miR-98 was simultaneously expressed during SARS-CoV-2 infection in vitro [59]. Additionally, miR-98-5p directly targets the 3’ untranslated region (UTR) of TMPRSS2, a gene involved in the viral entry and replication process in COVID-19 infection [60]. Another study reported downregulation of miR-98-5p in patients with moderate disease who were infected with COVID-19 [61].

MiR-98-5p, part of the let-7 family of miRNAs, is abnormally expressed in various cancers and plays crucial roles in cell proliferation, drug resistance, differentiation, metabolism, and angiogenesis. It targets inflammatory mediators like IL-6 and IL-10, though effects may vary by cell type [62]. Hypoxia-inducible factor-1α (HIF-1α), which is up-regulated under low oxygen and inflammatory conditions, can promote miR-98 expression. MiR-98 inhibits Factor Inhibiting HIF-1 (FIH-1), a negative regulator of HIF-1α, leading to HIF-1α activation and enhancing hypoxic responses. The relationship between miR-98 and HIF-1α is complex; miR-98 can both suppress HIF-1α and be upregulated by it under hypoxia, affecting inflammation and oxidative stress [63–65].

The elevated serum levels of miR-98-5p observed in our study may be attributed to a potential defensive mechanism employed by the body, particularly in cases involving prolonged hospitalization. Consistent with our findings, Liu D et al. and Liu C et al. reported that miR-98-5p is highly expressed in IgA nephropathy patients and targets chemokine ligand 3 (CCL3) to decrease its expression and increase the IL-6 level [66, 67].

Fujii reported that Cov-miR-5 produced from SARS-CoV-2 is a seed paralog of human miR-27a-3p with approximately 75% homology [34]. It was very difficult to determine Cov-miR-5 in our study. Therefore, we determined the expression levels of miR-27a-3p in the serum of COVID-19 patients, and our results were consistent with those of de Gonzalo-Calvo et al., who reported that miR-27a-3p was upregulated in ICU COVID-19 patients and that this upregulation was implicated in the regulation of immune and/or inflammatory pathways at different levels, such as T-cell development, differentiation, and activation [68].

Zhang et al. reported that miR-27a is an available miRNA that regulates drug targets for treating COVID-19 in clinical trials [8], as it can mediate the process of phagocytosis by regulating CD206 expression on monocytes [69].

All three studied miRs are strongly associated with female sex and are highly expressed in the 3rd wave. One of the possible explanations is that these miRs are related to the immune response, which is mostly prone to the female sex [70, 71]. Furthermore, as mentioned previously, the 3rd wave (Beta & Delta variants) may be more aggressive, highly transmissible, and able to cause more severe disease than the other variants of virus [46].

Research indicates that women generally demonstrate stronger cell-mediated and humoral immune responses and a higher susceptibility to autoimmune diseases compared to men [72]. Additionally, during chronic inflammation, women tend to have poorer prognoses and increased mortality, likely due to prolonged inflammation [73]. Moreover, autoimmune disorders occur more frequently in women [74], suggesting significant gender differences in immune system function and inflammatory responses between the sexes.

Finally, we emphasize the necessity of experimentally validating microRNAs in patient serum. Our findings reveal that the differential expression of miRs is robust and independent of the initial predictive model. This study demonstrates the successful translation of a bioinformatics-generated hypothesis into a verifiable biomarker through experimental validation, aligning with the aims of the multi-step approach.

## Conclusion

Finally, our study successfully converted an in silico generated theory into a real-world, testable biomarkers through laboratory experiments, proving their value even when the final experimental results didn’t match the initial theoretical predictions. In conclusion, the chosen microRNAs (miR-98-5p, miR-196a-5p, and miR-27-3p) could improve our understanding of the virus’s pathogenicity during various waves, indicating their potential as diagnostic or therapeutic biomarkers for SARS-CoV-2.

## Data Availability

The data analyzed or employed in the present work can be obtained from this work. Additional raw data may be available from the corresponding author upon request.
